# Hemodynamic and Clinical Predictors of Thrombolysis in Post-COVID Venous Thromboembolism: A Retrospective Cohort Study

**DOI:** 10.3390/biomedicines13092232

**Published:** 2025-09-10

**Authors:** Giulia-Mihaela Cojocaru, Antoniu Octavian Petriş, Alin-Constantin Pînzariu, Tudor Cojocaru, Andreea Coca, Ruxandra Cojocaru, Catherine-Teodora Costan, Victorița Șorodoc, Elena Cojocaru

**Affiliations:** 1 Faculty of Medicine, Doctoral School of Medicine, “Grigore T. Popa” University of Medicine and Pharmacy, 700115 Iasi, Romania; pinzariu_giulia@yahoo.com; 2 Medical I Department, Faculty of Medicine, “Grigore T. Popa” University of Medicine and Pharmacy, 700115 Iaşi, Romania; antoniu.petris@yahoo.ro; 3 Cardiology Clinic, “Sf. Spiridon” Clinical County Emergency Hospital, 700111 Iaşi, Romania; 4 Morpho-Functional Sciences II Department, Faculty of Medicine, “Grigore T. Popa” University of Medicine and Pharmacy, 700115 Iasi, Romania; elena.cojocaruu@umfiasi.ro; 5 Faculty of Medicine, “Grigore T. Popa” University of Medicine and Pharmacy, 700115 Iasi, Romania; tudorcoj@yahoo.com (T.C.); mg-rom-34267@students.umfiasi.ro (R.C.); mg-rom-34278@students.umfiasi.ro (C.-T.C.); 6 Department of Rheumatology, University of Pittsburgh Medical Center, Pittsburgh, PA 15213, USA; cocaa2@upmc.edu; 7 Internal Medicine and Toxicology Department, Faculty of Medicine, “Grigore T. Popa” University of Medicine and Pharmacy, 700115 Iasi, Romania; victorita.sorodoc@umfiasi.ro; 8 Medical III Department, Faculty of Medicine, “Grigore T. Popa” University of Medicine and Pharmacy, 700115 Iaşi, Romania

**Keywords:** COVID-19, echocardiography, post-COVID syndrome, pulmonary embolism, SpO_2_, TAPSE, thrombolysis, VTE

## Abstract

**Objectives:** Post-acute venous thromboembolism (VTE) is a well-recognized complication of COVID-19, driven by persistent endothelial dysfunction and thromboinflammation. Identifying simple clinical predictors of VTE may optimize therapy and limit adverse outcomes. We propose a pragmatic risk-stratification approach, based on clinical and echocardiographic parameters. **Methods:** We conducted a retrospective cohort study in a Romanian tertiary hospital (March 2020–April 2022) in 54 adults with laboratory-confirmed COVID-19 and imaging-confirmed VTE. Demographics, comorbidities, laboratory markers, and echocardiographic variables—particularly tricuspid annular plane systolic excursion (TAPSE), peripheral oxygen saturation (SpO_2_), and left-ventricular end-diastolic diameter (LVEDD)—were collected. The primary outcome was the percentage of patients receiving systemic thrombolysis. Statistical analyses included Mann–Whitney U tests, chi-square, Spearman correlations, and multivariable logistic regression. **Results:** The mean age was 61.2 ± 14.7 years, and 63% were men. Eleven patients (20.4%) underwent thrombolysis. Compared with conservatively managed patients, those receiving thrombolysis had lower TAPSE (13.0 vs. 20.8 mm), lower SpO_2_ (90.1 vs. 97.0%), and smaller LVEDD (24.4 vs. 46.1 mm); all differences were statistically significant. Each 1 mm decrease in TAPSE and 1% decrease in SpO_2_ increased the likelihood of thrombolysis (adjusted odds ratios 1.58 and 1.34, respectively). Inflammatory markers and right-ventricular diameter were not associated with treatment. **Conclusions:** Reduced TAPSE, lower SpO_2_, and decreased LVEDD identify post-COVID VTE patients at elevated risk of hemodynamic compromise requiring thrombolysis. A point-of-care assessment incorporating these variables may improve early risk stratification and guide therapeutic decisions.

## 1. Introduction

Venous thromboembolism (VTE), which includes pulmonary embolism (PE) and deep vein thrombosis (DVT), is the third leading cardiovascular cause of morbidity and mortality globally, after myocardial infarction and stroke [1]. Prior to the Coronavirus disease 2019 (COVID-19) pandemic, the incidence of VTE was estimated to be about 1 to 2 cases per 1,000 people per year in Western nations, with much lower rates in Eastern Europe. However, severe acute respiratory syndrome coronavirus 2 (SARS-CoV-2) has drastically changed this epidemiologic landscape [2]. VTE has been described with increasing frequency as a complication of COVID-19, particularly in critically ill patients, in whom the incidence of PE may be greater than 25%, despite prophylactic anticoagulation [3,4,5]. The prothrombotic mechanisms driven by the COVID-19 infection are further promoted by coagulation disorders secondary to innate and adaptive immune responses and endothelial dysfunction, leading to exaggerated immune activation, endothelial injury, and cytokine storm. Despite limited literature available on thromboembolic phenomena in pediatric patients with viral infections, mechanisms described—such as inflammation-mediated endothelial activation and coagulopathy—possess a generalized validity for any age range and are a useful background for contextualization of VTE in post-COVID patients [6,7].

**Figure 1:**
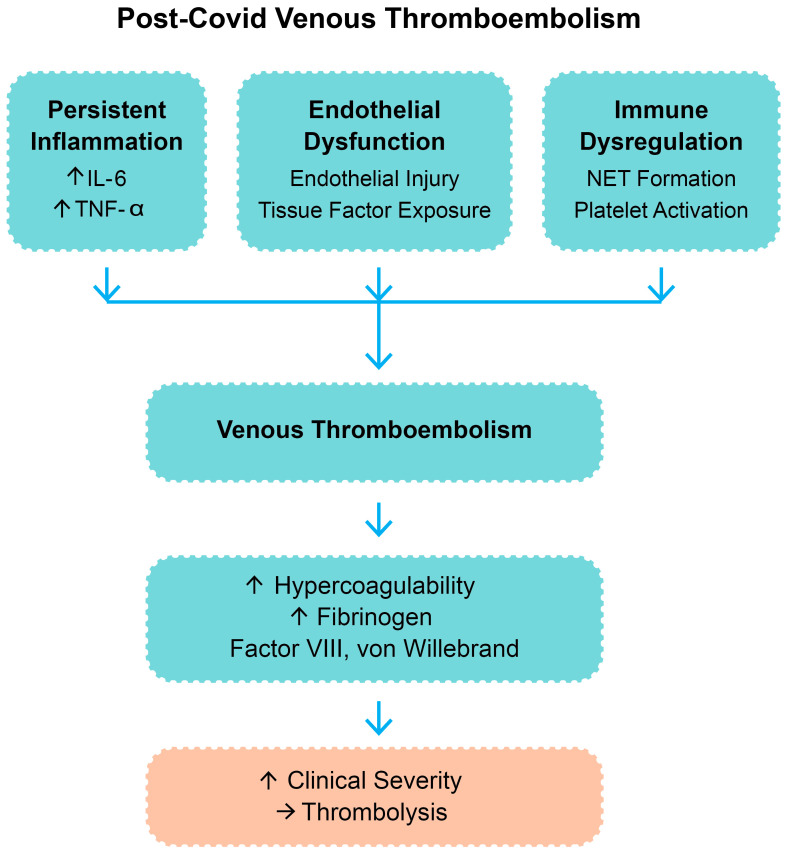
Pathophysiologic mechanisms contributing to post-COVID venous thromboembolism. Legend: IL-6—interleukin-6; TNF-α—tumor necrosis factor-alpha; NETs—neutrophil extracellular trap; “↑”—increase; “→”—result.

More recent evidence in adult cohorts has highlighted the contribution of SARS-CoV-2 to immunothrombosis through cytokine-driven platelet activation, complement system overactivation, and impaired fibrinolysis, all of which sustain a prothrombotic state even after the resolution of acute infection [1,2,5]. These overlapping processes support the rationale for considering thrombolysis in selected cases of post-COVID VTE. A summary of the main pathophysiological mechanisms is presented in Figure 1.

The pathophysiology of COVID-19-related VTE is intricate and multifactorial. SARS-CoV-2 binds to the angiotensin-converting enzyme 2 (ACE2) receptor, which is highly expressed on endothelial cells [8]. Viral entry and replication in the endothelium trigger endothelial damage that induces a prothrombotic phenotype by activation of the coagulation cascade and inhibition of fibrinolysis [9]. COVID-19 infection is also marked by an abnormal immune response that leads to a cytokine storm with elevated levels of interleukin-6 (IL-6), tumor necrosis factor-alpha (TNF-α), and other inflammatory mediators. Such cytokines upregulate the expression of tissue factor and cause platelet activation and aggregation [10,11,12]. Beyond COVID-19-associated coagulopathy, other underlying prothrombotic conditions—such as antiphospholipid syndrome and inherited thrombophilias—may also play a role in the pathogenesis of VTE in selected patients and warrant consideration [13].

In addition, neutrophil extracellular traps (NETs), network-like structures composed of deoxyribonucleic acid and cytotoxic proteins extracted by activated neutrophils, were identified in lung tissue and blood samples of COVID-19 patients [14,15]. NETs promote immunothrombosis and microvascular occlusion, which further compromise lung function [16,17]. The resulting coagulopathy is typically characterized by elevated D-dimer and fibrinogen levels, prolonged prothrombin time, and thrombocytopenia. This constellation of endothelial damage, hyperinflammation, and hypercoagulability is the aforementioned COVID-19-associated coagulopathy [18,19,20].

Although acute thrombotic complications during active infection are well described, recent reports have highlighted that the prothrombotic state can extend for weeks or even months following clinical recovery. Throughout this manuscript, “post-COVID” refers to the post-acute phase, defined as ≥10 days after confirmed SARS-CoV-2 infection, regardless of hospitalization status. The “post-COVID” period is one of residual endothelial activation and low-grade ongoing inflammation, both of which perpetuate thrombotic risk. Thus, rising numbers of patients are now presenting with VTE events in the post-acute COVID-19 period, with often no overt clinical predictors [21,22,23].

Within the context of PE, right ventricular (RV) function is a key determinant of clinical outcome. RV dysfunction may result in impaired forward flow, elevated right atrial pressures, and reduced left ventricular (LV) preload due to interventricular septal shift. Echocardiographic indices like tricuspid annular plane systolic excursion (TAPSE) yield an objective and reproducible assessment of RV systolic function. Decreased TAPSE has been associated with higher mortality in both PE and COVID-19 groups. Likewise, left ventricular underfilling, as evidenced by decreased LV end-diastolic diameter, can be an indirect sign of RV overload. Peripheral oxygen saturation (SpO_2_), a straightforward bedside measurement, can also indicate the degree of pulmonary vascular obstruction and gas exchange impairment [21,24,25].

Although echocardiography and pulse oximetry are widely accessible and frequently used in clinical practice, their combined application as structured tools for risk stratification in post-COVID venous thromboembolism remains insufficiently characterized in the current literature. In most clinical environments, especially in resource-scarce settings, the availability of advanced imaging techniques like computed tomography (CT) pulmonary angiography or invasive hemodynamic monitoring might be insufficient. Hence, the accessibility of readily available, non-invasive predictors of VTE severity and the requirement for intervention assume supreme significance.

Such overlapping mechanisms—including persistent inflammation, endothelial dysfunction, and immune dysregulation—promote a prothrombotic state that frequently extends beyond acute infection [26].

Figure 1 outlines the pathophysiologic pathways believed to be responsible for post-COVID venous thromboembolism and the rationale for thrombolytic therapy in carefully selected cases.

This work was designed to fill a knowledge gap by examining the correlation of echocardiographic (TAPSE, LV diameter, RV diameter) and clinical (SpO_2_) parameters along with thrombolysis utilization in post-COVID VTE patients. By evaluating a real-life cohort of patients who presented to an academic hospital, we seek to establish straightforward yet useful variables to stratify patient risk and aid decision management in this new clinical syndrome.

## 2. Materials and Methods

### 2.1. Study Design

The study was conducted at the Cardiology Clinic of the “Sf. Spiridon” Emergency Clinical Hospital, Iași, Romania, a university tertiary medical center. The study interval was March 2020 through April 2022, during several pandemic waves. Institutional ethics approval was obtained (Approval No. 46, 18 February 2025), and informed consent was waived due to the retrospective, anonymized nature of data collection.

### 2.2. Patient Selection

Patients were identified through hospital discharge records and the electronic medical registry. Eligible participants were adults (≥18 years) with a confirmed COVID-19 diagnosis, as evidenced by reverse transcriptase-polymerase chain reaction (RT-PCR) or confirmed rapid antigen testing, and a subsequent diagnosis of VTE, DVT, or PE, confirmed through imaging. Patients were included if the VTE occurred within 90 days of the confirmed SARS-CoV-2 infection.

### 2.3. Inclusion and Exclusion Criteria

Inclusion criteria comprised the following: age ≥18 years, confirmed SARS-CoV-2 infection by RT-PCR or antigen testing, imaged-confirmed diagnosis of PE (via CT pulmonary angiography) or DVT (via compression ultrasonography), transthoracic echocardiography performed within 24–48 h of VTE diagnosis, and availability of relevant laboratory values and clinical documentation. Exclusion criteria included chronic use of anticoagulant therapy prior to hospitalization for unrelated conditions (e.g., atrial fibrillation), diagnosis of active malignancy during the study period, incomplete clinical records or missing echocardiographic data, VTE diagnosis made more than 3 months post-COVID-19 infection, and duplicate or erroneous entries in the electronic health record.

### 2.4. Data Collection

Data was extracted independently by two trained researchers utilizing a standardized form. Data extracted were demographics (age, gender), comorbidities (hypertension, diabetes, obesity, chronic kidney disease, ischemic heart disease, COPD), admission vital signs (blood pressure, respiratory rate, SpO_2_), laboratory investigations (C-reactive protein, fibrinogen, creatinine, hemoglobin, D-dimer if available), and treatment variables (anticoagulant use, thrombolytic use).

### 2.5. Clinical and Echocardiographic Variables

Bedside transthoracic echocardiography was performed in all patients by senior cardiologists following a standard protocol on GE Vivid™ ultrasound systems. The following measurements were taken: TAPSE, taken using M-mode in apical 4-chamber view; RV basal diameter, taken in end-diastole at the greatest transverse dimension; and LV end-diastolic diameter (LVEDD), taken in parasternal long-axis view at end-diastole. All echocardiographic recordings were reviewed retrospectively and confirmed by a second independent cardiologist who was blinded to the patient’s outcome. Intra- and inter-observer agreement was evaluated in a random 20% sample and was >0.90 for all major variables.

The outcome of interest was the percentage of patients receiving systemic thrombolysis during hospitalization. Initiation of thrombolysis was at the discretion of the attending physician, according to clinical evaluation and bedside echocardiographic results. While management was generally in accordance with the European Society of Cardiology (ESC) 2019 guidelines, there was no formal institutional protocol for post-COVID VTE during the study interval.

Missing data were handled via case-wise deletion if essential fields (e.g., TAPSE or SpO_2_) were absent. For non-critical variables with less than 15% missingness, mean imputation was used. Outlier values were validated against original source documents. Categorical variables were coded, and continuous variables were tested for normality using the Shapiro-Wilk test. TAPSE was analyzed both as a continuous and categorical variable, using a cutoff of <17 mm to indicate RV dysfunction, in accordance with established echocardiographic guidelines [27].

### 2.6. Statistical Analysis

Descriptive statistics were expressed as mean ± standard deviation (SD) or median with interquartile range (IQR), as appropriate. The Shapiro-Wilk test was used to assess the normality of continuous variables. Continuous variables were compared using independent *t*-tests or Mann-Whitney U tests. Categorical variables were compared with the use of Pearson’s chi-square or Fisher’s exact test, as appropriate. The correlation between echocardiographic and clinical variables was evaluated with Spearman’s rank correlation. Logistic regression analysis was conducted to identify independent predictors of thrombolysis after adjustment for age, gender, and pertinent comorbidities. The association strength was expressed as odds ratios (OR) with 95% confidence intervals (CI). All analyses were performed using IBM SPSS Statistics version 26.0 (IBM Corp., Armonk, NY, USA) and R version 4.1.0 (R Foundation for Statistical Computing, Vienna, Austria). A *p*-value of <0.05 was regarded as statistically significant.

## 3. Results

A total of 54 patients met the inclusion criteria. The mean age of the cohort was 61.2 ± 14.7 years, with a male predominance (63%). Among the comorbidities, hypertension was the most common (59.3%), followed by type 2 diabetes mellitus (37.0%), obesity (31.5%), and chronic kidney disease (11.1%). All patients had confirmed COVID-19 infection and were diagnosed with VTE during the post-acute phase (median time from COVID-19 to VTE diagnosis: 12 days; IQR 6–24). Eleven patients (20.4%) received systemic thrombolytic therapy based on the clinical judgment and echocardiographic criteria described above. None of the patients in the cohort experienced in-hospital mortality, ICU admission, or bleeding complications following thrombolytic therapy.

Baseline clinical and laboratory characteristics of the study cohort are summarized in Table 1, which presents a comparative overview of demographic, respiratory, and echocardiographic parameters stratified by thrombolysis status.

Table 1 illustrates the differences between the two patient groups, particularly with regard to SpO_2_, TAPSE, and LV diameter. Patients who underwent thrombolysis exhibited mild hypotension during their admission, as evidenced by a comparative analysis of their systolic blood pressure levels with those in the control, suggesting early hemodynamic compromise despite preserved perfusion pressure. Also, the mean blood pressure reading was 127.3 mmHg, and the oxygen saturation level was found to be. These results indicate greater cardiorespiratory compromise on presentation in the thrombolysis group.

Thrombolysis group patients also had considerably lower TAPSE values compared to those who underwent conservative treatment. Peripheral oxygen saturation on admission was also considerably lower for the thrombolyzed patients compared to the non-thrombolysis group. LV end-diastolic diameter was reduced in the thrombolysis group, suggesting important interventricular interdependence. In contrast, no difference was noted between the groups in RV diameter.

Inflammatory and biochemical markers—like CRP, fibrinogen, and creatinine—did not show statistically significant differences between groups.

We retrospectively stratified each of the 54 patients in our dataset based on recognized high-risk VTE criteria to address variability in thrombolysis use. The simplified Pulmonary Embolism Severity Index (sPESI) was computed for each case after we retrieved hemodynamic and clinical characteristics (systolic and diastolic blood pressure, heart rate, oxygen saturation, presence of malignancy, chronic heart or lung illness, etc.) from the narrative summaries. Age > 80 years, active malignancy, chronic cardiopulmonary disease, heart rate ≥ 110 bpm, systolic blood pressure < 100 mmHg, and oxygen saturation < 90% all result in one point being awarded by the sPESI [28]. Presence of hemodynamic instability, which is defined as cardiac arrest, obstructive shock (systolic blood pressure < 90 mmHg or the need for vasopressors), or persistent hypotension (systolic blood pressure < 90 mmHg or a ≥ 40 mmHg drop lasting > 15 min), classified patients as “high risk” in accordance with European Society of Cardiology Guidelines (ESC) 2019 guidelines [29].

The retrospective application of these criteria showed that the patients who had thrombolysis were indeed at higher risk. Of the 11 thrombolysis cases, 8/11 satisfied the ESC criterion of hemodynamic instability, with a median sPESI of 3. By contrast, the median sPESI of the 43 non-thrombolysis patients was 1, and only 19 out of 43 met the ESC high-risk criterion. In other words, the considerably higher risk scores of the patients selected for thrombolysis demonstrate that physicians implicitly agreed with guideline-based criteria despite the absence of a clearly defined method. The distribution of sPESI scores and the percentage of high-risk patients in each category are summarized in Table 2. This post hoc adjudication makes the decision-making process more transparent and shows that the observed impact sizes are still significant even with a small sample.

Correlation analysis revealed TAPSE to be strongly inversely correlated with the probability of having received thrombolysis (Spearman ρ = −0.71, *p* < 0.001) and SpO_2_ to be inversely moderately correlated with thrombolysis (ρ = −0.49, *p* = 0.004). The LV diameter was moderately inversely correlated with thrombolytic administration (ρ = −0.41, *p* = 0.010). There was no significant correlation between RV diameter and any of the outcomes.

Logistic regression analysis demonstrated that both low TAPSE (OR 1.58 per mm decrease, 95% CI: 1.21–2.06; *p* = 0.001) and low SpO_2_ (OR 1.34 per % decrease, 95% CI: 1.08–1.66; *p* = 0.008) were independently associated with increased odds of receiving thrombolysis after adjustment for age, gender, hypertension, and diabetes. LV diameter was also predictive of thrombolysis (OR 0.91 per mm increase, 95% CI: 0.84–0.99; *p* = 0.032), which supports the fact that smaller LV dimensions were related to more severe hemodynamic compromise.

Further exploratory analyses stratified the cohort by TAPSE < 17 mm (n = 21) and TAPSE ≥ 17 mm (n = 33). Those with low TAPSE were more likely to receive thrombolysis (42.8% vs. 6.1%; *p* < 0.001), had lower SpO_2_ (89.5% vs. 96.5%, *p* = 0.009), and had smaller LV diameters (27.1 mm vs. 45.2 mm; *p* = 0.002). There were no differences in inflammatory markers between TAPSE subgroups.

**Figure 2:**
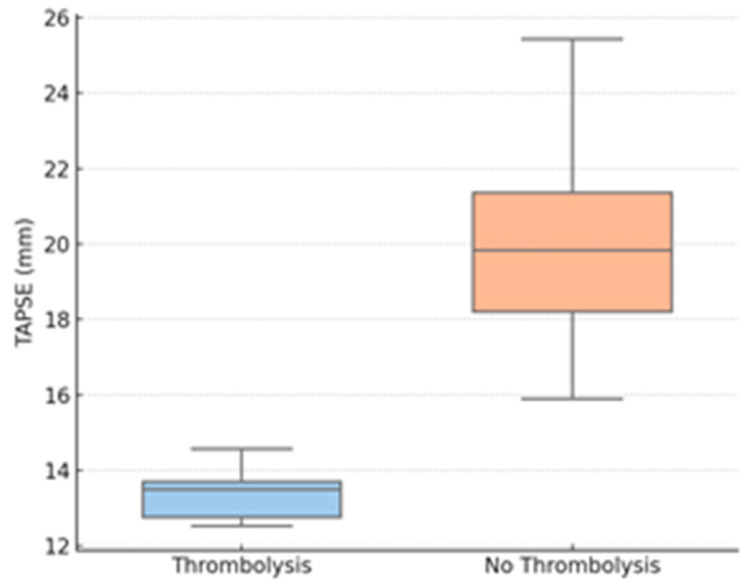
TAPSE by thrombolysis group. Legend: TAPSE—tricuspid annular plane systolic excursion.

**Figure 3:**
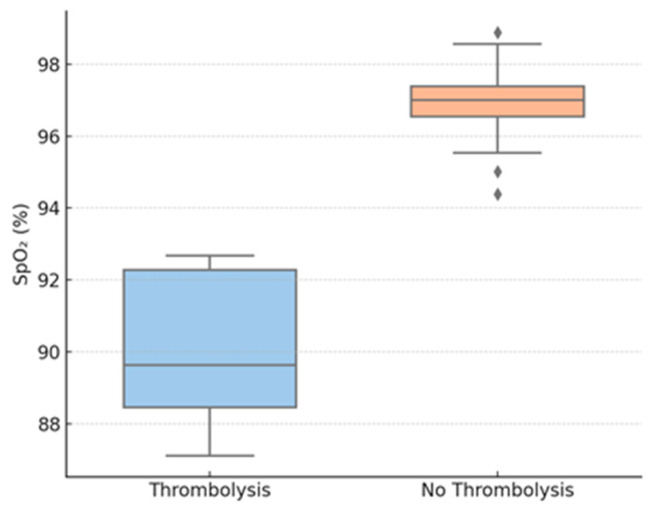
Oxygen saturation by thrombolysis group. Legend: SpO_2_—peripheral oxygen saturation.

**Figure 4:**
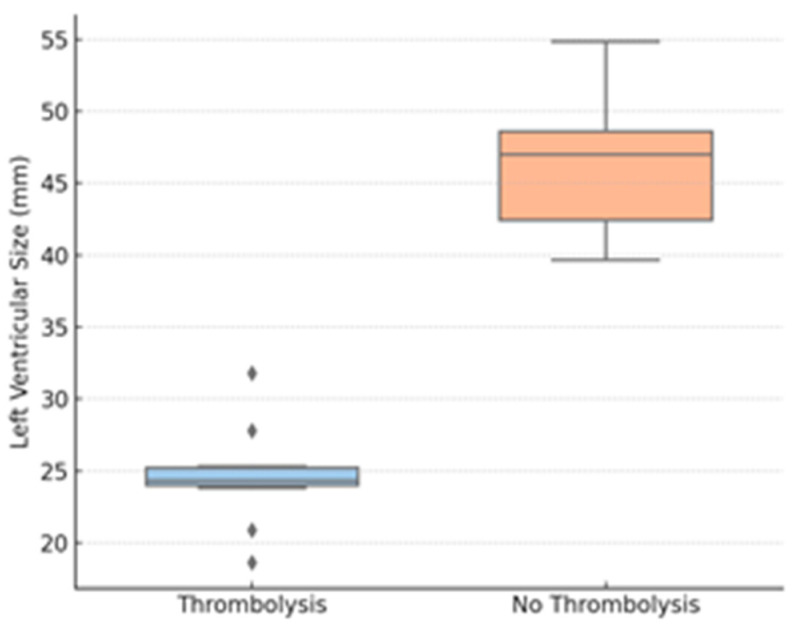
LV size by thrombolysis group. Legend: LVEDD—left ventricular end-diastolic diameter; diamonds represent outliers, defined as values outside 1.5 times the interquartile range (IQR).

The findings are plotted graphically over Figure 2, Figure 3 and Figure 4.

Figure 2 shows a boxplot of TAPSE values for thrombolysis vs. non-thrombolysis groups, demonstrating a significant decrease in RV function in the patients who needed intervention.

Figure 3 shows baseline SpO_2_, revealing a statistically significant reduction in the thrombolysis group.

Figure 4 shows the LVEDD, revealing interventricular dependence and reduced LV preload in more severe cases; diamonds represent outliers, defined as values outside 1.5 times the interquartile range (IQR).

Table 3 and Table 4 illustrate that among the variables evaluated, TAPSE has the most robust inverse correlation with the requirement for thrombolysis (Spearman ρ ≈ −0.71), i.e., increasingly lower TAPSE values are strongly related to an increased probability of thrombolytic therapy administration.

In summary, thrombolysis patients had worse oxygenation and echocardiographic profiles but comparable degrees of systemic inflammation. The cluster of low TAPSE, low SpO2, and small LV diameter seems to characterize a high-risk post-COVID VTE phenotype with considerable hemodynamic effect.

## 4. Discussion

### 4.1. Main Findings and Clinical Relevance

In this retrospective cohort study of post-COVID VTE, we show that patients that received thrombolytic therapy displayed certain echocardiographic and clinical characteristics. The strongest correlations with thrombolysis were decreases in TAPSE, SpO_2_, and LVEDD, suggesting their potential use as early noninvasive indicators of hemodynamic impairment. Spearman correlation analysis (Table 2) clarified the relative contributions of our variables: TAPSE demonstrated the strongest inverse association with the need for thrombolysis, whereas SpO_2_ and LVEDD showed only moderate inverse correlations. By contrast, age, CRP, fibrinogen, creatinine, and right-ventricular diameter were not significantly correlated with thrombolytic therapy, indicating that these commonly measured parameters offer limited predictive value in this setting. An exploratory assessment of relationships among TAPSE, SpO_2_, and LVEDD also revealed strongly positive correlations between these markers, suggesting they deteriorate in parallel in patients requiring escalated therapy; however, since all patients showed clinical improvement at discharge, no correlation with discharge status could be assessed. These findings have particular significance in the post-acute phase of COVID-19, when cardiac and thrombotic effects can persist even when inflammatory markers have returned to baseline. These results are particularly relevant because, in the post-acute phase of COVID-19, inflammatory markers may normalize, even as thrombotic and cardiovascular effects may persist.

### 4.2. Comparison with Previous Studies

A few studies have explored right ventricular function and oxygenation parameters in the setting of COVID-19-related thromboembolism [30,31]. Our observations are in line with these reports and additionally reinforce the clinical utility of echocardiographic and non-invasive markers to guide therapeutic decisions in post-COVID VTE. For instance, a large meta-analysis reported an overall prevalence of RV dysfunction of approximately 20%, which was significantly associated with increased mortality in hospitalized COVID-19 patients [32]. Additionally, a multicenter cohort study demonstrated that reduced TAPSE and impaired RV-arterial coupling were predictive of both pulmonary embolism and in-hospital mortality [31]. The ECHO–COVID study also reported common RV dysfunction among critically ill patients, with management being altered in as many as one-third of patients [33]. Single case reports also illustrate the diagnostic and prognostic utility of TAPSE and RV dilation in COVID-19 patients complicated by PE [34].

In our patient group, TAPSE values < 17 mm were highly predictive of thrombolysis administration, and all those with TAPSE < 13 mm received this therapy. This is also consistent with previous research that RV systolic dysfunction, evaluated by TAPSE or RV free wall strain, is correlated to higher mortality and intensive care unit (ICU) admission in COVID-19. Dweck et al. illustrated that RV enlargement and abnormal RV longitudinal strain were prevalent among hospitalized COVID-19 patients, especially in those who needed oxygen therapy, and were independently predictive of poor outcomes [35]. Likewise, Li et al. demonstrated that impaired TAPSE was associated with in-hospital mortality and the requirement for mechanical ventilation in COVID-19 pneumonia [36].

### 4.3. Physiological Rationale and Proposed Mechanism

The value of TAPSE as a straightforward, ubiquitous echocardiographic measure is well-suited to clinical triage, particularly where advanced imaging or hemodynamic monitoring is not available. Although strain imaging or the RV/LV ratio on CT angiography can assess RV size and function in detail, TAPSE serves as a practical and reproducible bedside alternative. Our work is one of the few to show that even mild decreases in TAPSE should be considered when evaluating the need for thrombolysis initiation in post-COVID patients, where submassive PE presentations may appear stable but carry a risk of sudden deterioration.

Another finding of our study relates to the clinical utility of SpO_2_ as a predictor of thrombolysis outcomes. While a ubiquitous measurement, the utility of SpO_2_ in VTE risk stratification has been understudied. In the analyzed cohort, SpO_2_ < 93% was strongly associated with the decision to initiate thrombolytic therapy. While hypoxemia in PE can be caused by ventilation-perfusion mismatch or increased dead space ventilation, it can also reflect reduced cardiac output due to right ventricular failure and interventricular dependence. Notably, desaturation occurs before the development of hypotension in submassive PE, thus representing an early sign of hemodynamic instability [37].

One of the most insightful observations in our cohort was the decreased LVEDD in thrombolysis patients. Left ventricular underfilling is a known effect of RV pressure overload and septal shift, especially in acute PE. This interventricular interdependence, where RV enlargement compromises LV diastolic compliance, can result in profound decreases in stroke volume and systemic perfusion, even among normotensive patients. This geometrical alteration, sometimes seen as a D-shaped left ventricle on echocardiogram, was indirectly measured in our cohort through LVEDD measurements. These findings are supported by previous experimental and clinical evidence showing decreased LVEDD to be related to elevated RV afterload and compromised cardiac output in PE. Importantly, this relationship has not been well characterized in post-COVID VTE, and our results indicate that LVEDD could be used as a surrogate for RV-LV interaction in this group [38].

**Figure 5:**
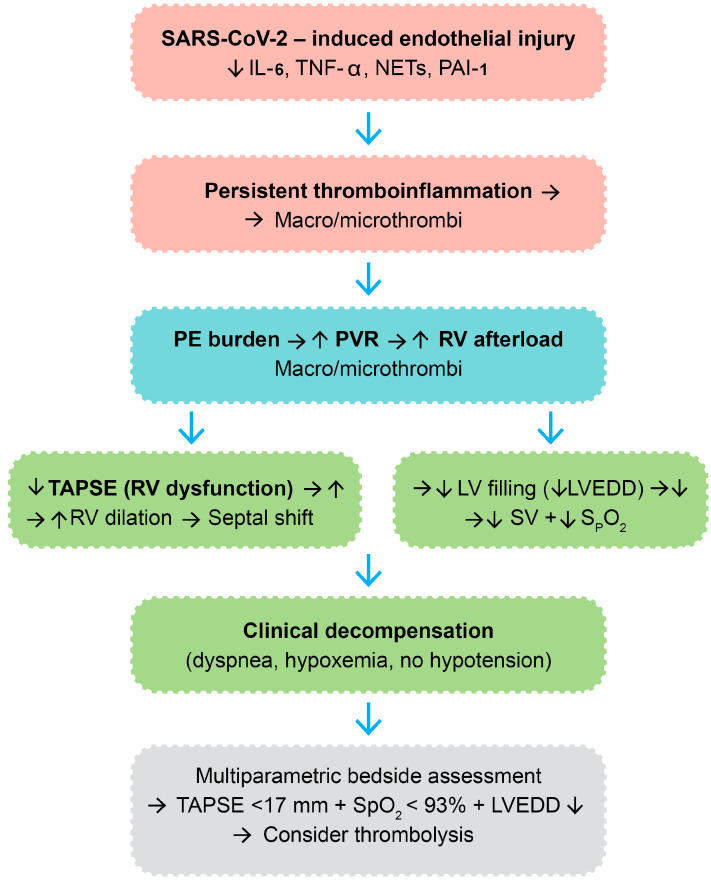
Integrated molecular and hemodynamic cascade in post-COVID venous thromboembolism leading to thrombolytic therapy. Legend: SARS-CoV-2—severe acute respiratory syndrome coronavirus 2; IL-6—interleukin-6; TNF-α—tumor necrosis factor-alpha; NETs—neutrophil extracellular traps; PAI-1—plasminogen activator inhibitor-1; PE—pulmonary embolism; PVR—pulmonary vascular resistance; RV—right ventricle; TAPSE—tricuspid annular plane systolic excursion; LV—left ventricle; LVEDD—left ventricle end-diastolic diameter; SV—stroke volume; SpO_2_—peripheral oxygen saturation; “→”—result; “↑”—increase; “↓”—decrease; color code: immune system—red, vascular system—blue, cardiac system—green.

A visual integration of the proposed pathophysiologic cascade—spanning from endothelial injury and thromboinflammation to hemodynamic collapse—is provided in Figure 5. This diagram illustrates the sequence of molecular and structural events culminating in clinical deterioration and therapeutic escalation in post-COVID VTE.

This pathophysiologic framework theoretically delineates the molecular and hemodynamic cascade whereby SARS-CoV-2 infection can culminate in VTE and other clinical decompensation necessitating thrombolytic therapy in a subset of post-COVID patients. The cascade is initiated by viral binding to ACE2 receptors of vascular endothelial cells, with resultant direct endothelial damage and apoptosis [39]. This results in endothelial activation with upregulation of the adhesion molecules (ICAM-1, VCAM-1, and E-selectin) and causes Weibel-Palade body exocytosis, platelet adherence, and leukocyte recruitment [40,41]. Concurrently, infected and activated endothelial cells and immune cells release a spectrum of pro-inflammatory cytokines, including IL-6, TNF-α, and IL-1β, and chemokines like chemokine (CXC motif) ligand 8-CXCL8, causing local and systemic inflammation [42,43,44]. The endothelium also secretes plasminogen activator inhibitor-1 (PAI-1), which inhibits fibrinolysis, and monocytes and macrophages upregulate tissue factor, activating the extrinsic coagulation cascade [45,46].

The ongoing inflammatory milieu, along with immune dysregulation, stimulates a pro-thrombotic environment. NETs, which are generated via NETosis, serve as a scaffold for coagulation, bind factor XII, and trigger the intrinsic coagulation cascade, with enhancement of thrombin generation [47]. This hypercoagulable state leads to the formation of macrovascular thrombi—i.e., deep vein thrombosis or pulmonary embolism—and microvascular thrombi, particularly in the pulmonary capillaries and right heart vasculature, often without obvious systemic hypotension.

The hemodynamic burden of pulmonary embolic obstruction is an acute rise in pulmonary vascular resistance (PVR) that acutely increases RV afterload. This raises RV wall stress, thus uncoupling RV-arterial coupling. RV oxygen demand rises, and perfusion mismatches and capillary occlusion compromise the oxygen supply to the myocardium, paving the way for RV ischemia and progressive systolic dysfunction [48].

Echocardiographically, RV dysfunction would typically present as a reduction in TAPSE, reflecting impaired longitudinal RV contractility. Chronic pressure overload leads to RV dilation and interventricular septal bowing toward the LV, also described as the D-sign. This geometric distortion creates interventricular dependence, in which left ventricular diastolic filling is impaired. Beyond structural changes, at the myocyte level, deranged calcium handling and mitochondrial dysfunction may further compromise RV contractility [49].

The consequent left ventricular underfilling can be quantified as a reduction in LVEDD. In the setting of preserved systolic function, preload restriction lowers stroke volume and systemic cardiac output [50]. Concurrently, pulmonary perfusion-ventilation mismatching and impaired CO_2_ clearance cause systemic arterial hypoxemia, which presents as reduced SpO_2_.

### 4.4. Clinical Implications for Thrombolytic Decision-Making

Although classical signs of massive pulmonary embolism—such as systemic hypotension or syncope—may not be present, this constellation of RV dysfunction, LV underfilling, and hypoxemia frequently leads to clinical decompensation. Patients often present with progressive dyspnea, tachycardia, and oxygen desaturation, despite preserved blood pressure. Inflammatory biomarkers can be raised at this point but provide relatively nonspecific information for guiding therapy. In this setting, a multiparametric bedside evaluation is imperative. The concurrence of decreased TAPSE (<17 mm), low SpO_2_ (<93%), and decreased LVEDD is a reproducible and practical constellation of findings indicative of hemodynamic compromise in the absence of overt shock. This phenotype, which is especially common in post-COVID patients with ongoing endothelial dysfunction, can help identify high-risk patients with potential for progression who may benefit from early thrombolytic therapy to prevent irreversible RV failure.

Overall, this model stresses the interaction between molecular inflammation, coagulation, cardiopulmonary mechanics, and clinical decompensation in post-COVID VTE. It calls for a systematic, bedside-driven strategy that considers structural echocardiographic parameters in addition to functional oxygenation indices. This integrative approach enables the identification of a high-risk, submassive VTE phenotype early on, with the aim of prompt and personalized therapeutic management in a cohort at risk of silent worsening and adverse outcomes.

Our study also examined the performance of traditional inflammatory and thrombotic biomarkers such as CRP and fibrinogen. While both are frequently elevated in acute COVID-19 infection and are thought to mediate immunothrombosis, they were not significantly associated with thrombolysis initiation in our cohort. This observation could reflect the interval between SARS-CoV-2 infection onset and the ensuing thromboembolic event in our patient group, during which systemic inflammation may have resolved. Furthermore, this finding underscores the potential limitations of sole reliance on biochemical markers in post-acute COVID-19 settings and supports the use of dynamic functional markers, including echocardiography and oxygenation parameters. Zuo et al. have already emphasized that NETs, rather than CRP or D-dimer alone, could have a central role in COVID-19-mediated microvascular thrombosis [51]; unfortunately, NETs are not generally quantifiable in the routine clinical setting.

Relative to prior work, our study has several novel aspects. First, although other authors have reported RV strain or dilation in acute COVID-19, few have explored echocardiographic parameters in the setting of post-COVID VTE, in which patients often present with late-onset PE, frequently without overt signs. Second, our emphasis on real-world thrombolysis decisions—as opposed to surrogate endpoints like ICU admission or mortality—yields pragmatic lessons regarding bedside evaluation and therapy escalation. Third, our study identifies a straightforward, reproducible triad (TAPSE < 17 mm, SpO_2_ < 93%, reduced LVEDD) that, in a clinical setting, may aid clinicians in determining high-risk patients prior to hemodynamic collapse. In environments without readily available CT pulmonary angiography or invasive hemodynamic monitoring, this triad could represent an important adjunct for early therapeutic decisions.

Our study provides evidence of the clinical usefulness of non-invasive bedside markers—TAPSE, SpO_2_, and LVEDD—in the recognition of patients with post-COVID VTE who are likely to develop hemodynamic compromise. These easily measured parameters, which can be derived from echocardiography and pulse oximetry, can potentially guide therapeutic escalation even without the presence of hypotension. Their incorporation into early risk stratification protocols can improve patient safety, rationalize resource utilization, and improve outcomes in this challenging and increasing group of patients. Notably, in our cohort, thrombolytic therapy was not associated with any cases of in-hospital death, ICU transfer, or bleeding events.

### 4.5. Limitations

This study has some limitations. A central methodological limitation of this study is the retrospective study design and modest sample size, especially in the thrombolysis subgroup (n = 11). These characteristics may constrain statistical power and heighten the risk of type II error. Notably, systemic thrombolysis was initiated based on clinical judgment and not according to standardized institutional protocols. Although general principles were consistent with the ESC 2019 pulmonary embolism guidelines, precise criteria for post-COVID VTE were not institutionalized during the period of study. This has the potential to introduce selection bias; however, it also captures real-world decision-making in the context of clinical uncertainty and emerging evidence. Also, certain key risk stratification tools, like troponin, N-terminal pro-B-type natriuretic peptide, or validated score systems (e.g., Pulmonary Embolism Severity Index—PESI or BOVA), were not regularly documented and therefore not included in the analysis. To address sample size limitations, we performed a post hoc power calculation based on values taken from clinical data. Both comparisons between thrombolysis and non-thrombolysis groups for TAPSE and oxygen saturation were large (Cohen’s d > 1.5) and had > 80% power, thus in favor of clinical relevance. Small blood pressure differences had low power (<20%), which should be interpreted with caution. Because of the real-world and retrospective nature of the dataset, more predictive modeling and comorbidity subgroup analysis could not be performed reliably. Lastly, there was no long-term follow-up data with which to evaluate survival, recurrence, or bleeding complications after thrombolytic therapy.

Furthermore, it is important to consider how biological samples can help refine treatment choices.

### 4.6. Future Directions

Since increased thrombin generation parameters could be another indicator for thrombolysis—even in the absence of right ventricular dysfunction—a prospective study evaluating thrombin generation in individuals with post-COVID VTE may be beneficial. Although the high cost of reagents, difficulty of standardization, and requirement for specialized coagulometers prevent thrombin production assays from being used frequently, their inclusion in future studies may help improve clinical results and patient selection for thrombolytic therapy.

Our findings suggest that the combination of low TAPSE, reduced SpO_2_, and smaller LV end-diastolic diameter identifies a distinct hemodynamic phenotype of post-COVID VTE. Although each parameter individually showed moderate-to-strong correlation with the need for thrombolysis, their simultaneous alteration may help recognize patients at risk of decompensation even in the absence of hypotension. This cluster may be particularly useful for early risk stratification at the bedside, when other advanced tools are unavailable.

Future prospective multicenter studies are needed to validate these findings, incorporate more comprehensive hemodynamic and biochemical markers, and establish standard protocols for therapeutic escalation in post-COVID VTE.

## 5. Conclusions

This study identified a non-invasive, multiparametric profile—consisting of decreased TAPSE, SpO_2_, and LVEDD—that is powerfully linked to the requirement for thrombolysis in post-COVID VTE. In contrast to previous research focused on acute COVID or individual markers—such as elevated D-dimer or isolated RV/LV ratio on CT angiography—our results highlight a physiologically integrated strategy for early risk stratification, even among normotensive patients. This model has the potential to be especially useful in resource-scarce environments and deserves prospective validation for inclusion in future clinical decision-making algorithms.

## Figures and Tables

**Table 1:** Baseline clinical and imaging parameters by thrombolysis status.

Parameter	Thrombolysis (n = 11)	No Thrombolysis (n = 43)	*p*-Value
Age, years (mean ± SD)	62.5 ± 13.1	60.9 ± 15.0	0.71
Male gender, n (%)	7 (63.6)	27 (62.8)	0.96
Systolic BP, mmHg (mean ± SD)	119.6 ± 26.0	129.3 ± 19.0	0.042
Diastolic BP, mmHg (mean ± SD)	73.3 ± 13.2	78.4 ± 13.4	0.27
SpO_2_ on admission, % (mean ± SD)	90.1 ± 5.1	97.0 ± 2.8	0.013 *
TAPSE, mm (mean ± SD)	13.0 ± 2.5	20.8 ± 5.1	<0.001 *
LV diameter, mm (mean ± SD)	24.4 ± 6.2	46.1 ± 8.9	0.009 *
RV diameter, mm (mean ± SD)	34.8 ± 6.0	33.6 ± 7.3	0.87
CRP, mg/L (mean ± SD)	117.4 ± 64.3	105.7 ± 58.8	0.71
Fibrinogen, mg/dL (median)	515	525	0.86
Creatinine, mg/dL (mean ± SD)	1.13 ± 0.26	1.08 ± 0.31	0.49

* Statistically significant. Legend: n—number; *p*—probability value; SD—standard deviation; BP—blood pressure; SpO—peripheral oxygen saturation; TAPSE—tricuspid annular plane systolic excursion; LV—left ventricle; RV—right ventricle; CRP—C-reactive protein.

**Table 2:** Risk profile by treatment group.

Group	Number of Patients (n)	Median sPESI (Mean ± SD)	Patients with ESC High-Risk Criteria	Notes
Thrombolysis	11	3 (2.27 ± 1.4)	8/11 (73%)	Higher frequency of systolic BP < 90 mmHg and hypoxemia
No thrombolysis	43	1 (1.58 ± 1.3)	19/43 (44%)	15/43 (35%) had hypoxemia without hypotension

Legend: n—number; sPESI—simplified Pulmonary Embolism Severity Index; SD—standard deviation; ESC—European Society of Cardiology; BP—blood pressure.

**Table 3:** Univariate analysis—correlation with thrombolysis.

Variable	Spearman ρ (vs. Thrombolysis)	Observations
TAPSE (mm)	−0.71	Strong inverse correlation; lower TAPSE increases the likelihood of thrombolysis.
SpO_2_ on admission (%)	−0.49	Moderate inverse correlation; lower oxygen saturation increases the risk of thrombolysis.
LV end-diastolic diameter (mm)	−0.41	Moderate inverse correlation; a smaller left ventricular diameter is associated with thrombolysis.
RV diameter (mm)	≈0 (not significant)	No significant correlation with thrombolysis.

Legend: mm—millimeters; TAPSE—tricuspid annular plane systolic excursion; SpO_2_—peripheral oxygen saturation; LV—left ventricle; RV—right ventricle.

**Table 4:** Multivariate analysis—logistic regression predictors.

Variable	Odds Ratio per Unit (95% CI)	Interpretations
TAPSE (mm)	1.58 per mm decrease (1.21–2.06)	Lower TAPSE is an independent predictor of thrombolysis.
SpO_2_ on admission (%)	1.34 per % decrease (1.08–1.66)	Lower oxygen saturation is an independent predictor of thrombolysis.
LV end-diastolic diameter (mm)	0.91 per mm increase (0.84–0.99)	Smaller LV diameter is independently associated with thrombolysis.
RV diameter (mm)	Not significant	Not a significant independent predictor

Legend: *p*—probability value; CI—confidence interval; TAPSE—tricuspid annular plane systolic excursion; SpO_2_—peripheral oxygen saturation; LV—left ventricle; RV—right ventricle.

## Data Availability

The data presented in this study are not publicly available due to privacy and ethical restrictions.

## References

[1] Raskob G.E., Angchaisuksiri P., Blanco A.N., Buller H., Gallus A., Hunt B.J., Hylek E.M., Kakkar A., Konstantinides S.V., McCumber M. (2014). Thrombosis: A major contributor to global disease burden. Arterioscler. Thromb. Vasc. Biol..

[2] Kearon C. (2001). Epidemiology of venous thromboembolism. Semin. Vasc Med..

[3] Boyd S., Martin-Loeches I. (2021). The incidence of venous thromboembolism in critically ill patients with COVID-19 compared with critically ill non-COVID patients. Ir. J. Med. Sci..

[4] Landi A., De Servi S. (2020). The burden of thrombotic complications in critically ill patients with COVID-19: Charting the uncharted. Intern. Emerg. Med..

[5] Boyd S., Sheng Loh K., Lynch J., Alrashed D., Muzzammil S., Marsh H., Masoud M., Bin Ihsan S., Martin-Loeches I. (2022). The Incidence of Venous Thromboembolism in Critically Ill Patients with SARS-CoV-2 Infection Compared with Critically Ill Influenza and Community-Acquired Pneumonia Patients: A Retrospective Chart Review. Med. Sci..

[6] Lassandro G., Palladino V., Amoruso A., Palmieri V.V., Russo G., Giordano P. (2020). Children in Coronaviruses’ Wonderland: What Clinicians Need to Know. Mediterr. J. Hematol. Infect. Dis..

[7] Lassandro G., Palmieri V.V., Palladino V., Amoruso A., Faienza M.F., Giordano P. (2020). Venous Thromboembolism in Children: From Diagnosis to Management. Int. J. Environ. Res. Public Health.

[8] Mezoh G., Crowther N.J. (2021). Endothelial Dysfunction as a Primary Consequence of SARS-CoV-2 Infection. Adv. Exp. Med. Biol..

[9] Six I., Guillaume N., Jacob V., Mentaverri R., Kamel S., Boullier A., Slama M. (2022). The Endothelium and COVID-19: An Increasingly Clear Link Brief Title: Endotheliopathy in COVID-19. Int. J. Mol. Sci..

[10] Shekhawat J., Gauba K., Gupta S., Purohit P., Mitra P., Garg M., Misra S., Sharma P., Banerjee M. (2021). Interleukin-6 Perpetrator of the COVID-19 Cytokine Storm. Indian J. Clin. Biochem..

[11] Faraj S.S., Jalal P.J. (2023). IL1β, IL-6, and TNF-α cytokines cooperate to modulate a complicated medical condition among COVID-19 patients: Case-control study. Ann. Med. Surg..

[12] Paranga T.G., Mitu I., Pavel-Tanasa M., Rosu M.F., Miftode I.L., Constantinescu D., Obreja M., Plesca C.E., Miftode E. (2024). Cytokine Storm in COVID-19: Exploring IL-6 Signaling and Cytokine-Microbiome Interactions as Emerging Therapeutic Approaches. Int. J. Mol. Sci..

[13] Evangelidis P., Kotsiou N., Kalmoukos P., Ntova Z., Papadopoulou T., Chissan S., Sarvani A., Kokoris S., Grouzi E., Doumas M. (2025). Prevalence and Risk Factors of Acute Ischemic Stroke in Patients with Antiphospholipid Syndrome: A Retrospective Monocenter Analysis. J. Cardiovasc. Dev. Dis..

[14] Li S., Wang H., Shao Q. (2023). The central role of neutrophil extracellular traps (NETs) and by-products in COVID-19 related pulmonary thrombosis. Immun. Inflamm. Dis..

[15] Radermecker C., Detrembleur N., Guiot J., Cavalier E., Henket M., d’Emal C., Vanwinge C., Cataldo D., Oury C., Delvenne P. (2020). Neutrophil extracellular traps infiltrate the lung airway, interstitial, and vascular compartments in severe COVID-19. J. Exp. Med..

[16] Wang H., Kim S.J., Lei Y., Wang S., Wang H., Huang H., Zhang H., Tsung A. (2024). Neutrophil extracellular traps in homeostasis and disease. Signal Transduct. Target. Ther..

[17] Ackermann M., Anders H.J., Bilyy R., Bowlin G.L., Daniel C., De Lorenzo R., Egeblad M., Henneck T., Hidalgo A., Hoffmann M. (2021). Patients with COVID-19: In the dark-NETs of neutrophils. Cell Death Differ..

[18] Dubey L., Dorosh O., Dubey N., Doan S., Kozishkurt O., Duzenko O., Kozlova O., Ievtukh V., Ladny J.R., Pruc M. (2023). COVID-19-induced coagulopathy: Experience, achievements, prospects. Cardiol. J..

[19] Lorini F.L., Di Matteo M., Gritti P., Grazioli L., Benigni A., Zacchetti L., Bianchi I., Fabretti F., Longhi L. (2021). Coagulopathy and COVID-19. Eur. Heart J. Suppl. J. Eur. Soc. Cardiol..

[20] Iba T., Connors J.M., Levy J.H. (2020). The coagulopathy, endotheliopathy, and vasculitis of COVID-19. Inflamm. Res. Off. J. Eur. Histamine Res. Soc..

[21] Boccatonda A., Campello E., Simion C., Simioni P. (2023). Long-term hypercoagulability, endotheliopathy and inflammation following acute SARS-CoV-2 infection. Expert Rev. Hematol..

[22] Martins-Gonçalves R., Hottz E.D., Bozza P.T. (2023). Acute to post-acute COVID-19 thromboinflammation persistence: Mechanisms and potential consequences. Curr. Res. Immunol..

[23] Giaglis S. (2023). Poised to cast wide NETs in long COVID. J. Thromb. Haemost..

[24] Ro S.K., Sato K., Ijuin S., Sela D., Fior G., Heinsar S., Kim J.Y., Chan J., Nonaka H., Lin A.C.W. (2023). Assessment and diagnosis of right ventricular failure-retrospection and future directions. Front. Cardiovasc. Med..

[25] Monitillo F., Di Terlizzi V., Gioia M.I., Barone R., Grande D., Parisi G., Brunetti N.D., Iacoviello M. (2020). Right Ventricular Function in Chronic Heart Failure: From the Diagnosis to the Therapeutic Approach. J. Cardiovasc. Dev. Dis..

[26] Butcovan D., Oboroceanu T., Cimpeanu C., Mironescu A., Haliga R.E., Pinzariu A.C., Lupusoru R.V., Popescu E., Mocanu V. (2017). The Involvement of Epicardial Adiposity and Inflammation in Postoperatory Atrial Fibrilation–Immunohistochemical Qualitative and Quantitative Assessment. RevChim.

[27] Lang R.M., Badano L.P., Mor-Avi V., Afilalo J., Armstrong A., Ernande L., Flachskampf F.A., Foster E., Goldstein S.A., Kuznetsova T. (2015). Recommendations for cardiac chamber quantification by echocardiography in adults: An update from the American Society of Echocardiography and the European Association of Cardiovascular Imaging. Eur. Heart J. Cardiovasc. Imaging.

[28] Jiménez D. Simplified PESI (Pulmonary Embolism Severity Index). MDCalc. https://www.mdcalc.com/calc/1247/simplified-pesi-pulmonary-embolism-severity-index.

[29] Pérez-Nieto O.R., Gómez-Oropeza I., Quintero-Leyra A., Kammar-García A., Zamarrón-López É.I., Soto-Estrada M., Morgado-Villaseñor L.A., Meza-Comparán H.D. (2023). Hemodynamic and respiratory support in pulmonary embolism: A narrative review. Front. Med..

[30] Bonnemain J., Ltaief Z., Liaudet L. (2021). The Right Ventricle in COVID-19. J. Clin. Med..

[31] Polito M.V., Silverio A., Di Maio M., Bellino M., Scudiero F., Russo V., Rasile B., Alfano C., Citro R., Parodi G. (2021). Prognostic Implications of Right Ventricular Function and Pulmonary Pressures Assessed by Echocardiography in Hospitalized Patients with COVID-19. J. Pers. Med..

[32] Corica B., Marra A.M., Basili S., Cangemi R., Cittadini A., Proietti M., Romiti G.F. (2021). Prevalence of right ventricular dysfunction and impact on all-cause death in hospitalized patients with COVID-19: A systematic review and meta-analysis. Sci. Rep..

[33] Huang S., Vignon P., Mekontso-Dessap A., Tran S., Prat G., Chew M., Balik M., Sanfilippo F., Banauch G., Clau-Terre F. (2022). Echocardiography findings in COVID-19 patients admitted to intensive care units: A multi-national observational study (the ECHO-COVID study). Intensive Care Med..

[34] Ullah W., Saeed R., Sarwar U., Patel R., Fischman D.L. (2020). COVID-19 Complicated by Acute Pulmonary Embolism and Right-Sided Heart Failure. JACC Case Rep..

[35] Dweck M.R., Bularga A., Hahn R.T., Bing R., Lee K.K., Chapman A.R., White A., Di Salvo G., Sade L.E., Pearce K. (2020). Global evaluation of echocardiography in patients with COVID-19. Eur. Heart J. Cardiovasc. Imaging.

[36] Li Y.L., Zheng J.B., Jin Y., Tang R., Li M., Xiu C.H., Dai Q.-Q., Zuo S., Wang H.-Q., Wang H.-L. (2020). Acute right ventricular dysfunction in severe COVID-19 pneumonia. Rev. Cardiovasc. Med..

[37] Konstantinides S.V., Meyer G., Becattini C., Bueno H., Geersing G.J., Harjola V.P., Huisman M.V., Humbert M., Jennings C.S., Jiménez D. (2020). 2019 ESC Guidelines for the diagnosis and management of acute pulmonary embolism developed in collaboration with the European Respiratory Society (ERS). Eur. Heart J..

[38] Goldhaber S.Z., Elliott C.G. (2003). Acute pulmonary embolism: Part I: Epidemiology, pathophysiology, and diagnosis. Circulation.

[39] Huertas A., Montani D., Savale L., Pichon J., Tu L., Parent F., Guignabert C., Humbert M. (2020). Endothelial cell dysfunction: A major player in SARS-CoV-2 infection (COVID-19)?. Eur. Respir. J..

[40] Kim I., Moon S.O., Kim S.H., Kim H.J., Koh Y.S., Koh G.Y. (2001). Vascular endothelial growth factor expression of intercellular adhesion molecule 1 (ICAM-1), vascular cell adhesion molecule 1 (VCAM-1), and E-selectin through nuclear factor-kappa B activation in endothelial cells. J. Biol. Chem..

[41] Schillemans M., Karampini E., Kat M., Bierings R. (2019). Exocytosis of Weibel-Palade bodies: How to unpack a vascular emergency kit. J. Thromb. Haemost..

[42] Kany S., Vollrath J.T., Relja B. (2019). Cytokines in Inflammatory Disease. Int. J. Mol. Sci..

[43] Cambier S., Gouwy M., Proost P. (2023). The chemokines CXCL8 and CXCL12: Molecular and functional properties, role in disease and efforts towards pharmacological intervention. Cell Mol. Immunol..

[44] Scheller J., Chalaris A., Schmidt-Arras D., Rose-John S. (2011). The pro- and anti-inflammatory properties of the cytokine interleukin-6. Biochim. Biophys. Acta.

[45] Levi M., Keller T.T., van Gorp E., ten Cate H. (2003). Infection and inflammation and the coagulation system. Cardiovasc. Res..

[46] Whyte C.S. (2023). All tangled up: Interactions of the fibrinolytic and innate immune systems. Front. Med..

[47] de Bont C.M., Boelens W.C., Pruijn G.J.M. (2019). NETosis, complement, and coagulation: A triangular relationship. Cell Mol. Immunol..

[48] Verma N., Kondoor V., Singh R., Ahuja R. (2025). Acute RV Failure Management in Pulmonary Embolism. Tech. Vasc. Interv. Radiol..

[49] Zaidi A., Knight D.S., Augustine D.X., Harkness A., Oxborough D., Pearce K., Ring L., Robinson S., Stout M., Willis J. (2020). Echocardiographic assessment of the right heart in adults: A practical guideline from the British Society of Echocardiography. Echo Res. Pract..

[50] Jozwiak M., Teboul J.L. (2024). Heart-Lungs interactions: The basics and clinical implications. Ann. Intensive Care.

[51] Zuo Y., Yalavarthi S., Shi H., Gockman K., Zuo M., Madison J.A., Blair C., Weber A., Barnes B.J., Egeblad M. (2020). Neutrophil extracellular traps in COVID-19. JCI Insight.

